# Macrophage Selenoproteins Restrict Intracellular Replication of *Francisella tularensis* and Are Essential for Host Immunity

**DOI:** 10.3389/fimmu.2021.701341

**Published:** 2021-10-29

**Authors:** Rachel L. Markley, Katherine H. Restori, Bhuvana Katkere, Sarah E. Sumner, McKayla J. Nicol, Anastasia Tyryshkina, Shaneice K. Nettleford, David R. Williamson, David E. Place, Kalyan K. Dewan, Ashley E. Shay, Bradley A. Carlson, Santhosh Girirajan, K. Sandeep Prabhu, Girish S. Kirimanjeswara

**Affiliations:** ^1^Pathobiology Graduate Program, The Pennsylvania State University, University Park, PA, United States; ^2^Department of Veterinary and Biomedical Sciences, The Pennsylvania State University, University Park, PA, United States; ^3^Department of Cardiovascular and Metabolic Sciences, Lerner Research Institute, Cleveland Clinic, Cleveland, OH, United States; ^4^Neuroscience Graduate Program, Huck Institute of the Life Sciences, The Pennsylvania State University, University Park, PA, United States; ^5^Department of Biochemistry and Molecular Biology, The Pennsylvania State University, University Park, PA, United States; ^6^Department of Immunology, St. Jude Children’s Research Hospital, Memphis, TN, United States; ^7^Department of Infectious Diseases, The University of Georgia, Athens, GA, United States; ^8^Center for Experimental Therapeutics and Reperfusion Injury, Department of Anesthesiology, Perioperative and Pain Medicine, Brigham and Women’s Hospital and Harvard Medical School, Boston, MA, United States; ^9^Office of Research Support, Center for Cancer Research, National Cancer Institute, National Institutes of Health, Bethesda, MD, United States; ^10^Center for Molecular Immunology and Infectious Disease, The Pennsylvania State University, University Park, PA, United States

**Keywords:** selenium, redox, intracellular bacteria, tularemia, innate immunity

## Abstract

The essential micronutrient Selenium (Se) is co-translationally incorporated as selenocysteine into proteins. Selenoproteins contain one or more selenocysteines and are vital for optimum immunity. Interestingly, many pathogenic bacteria utilize Se for various biological processes suggesting that Se may play a role in bacterial pathogenesis. A previous study had speculated that *Francisella tularensis*, a facultative intracellular bacterium and the causative agent of tularemia, sequesters Se by upregulating Se-metabolism genes in type II alveolar epithelial cells. Therefore, we investigated the contribution of host vs. pathogen-associated selenoproteins in bacterial disease using *F. tularensis* as a model organism. We found that *F. tularensis* was devoid of any Se utilization traits, neither incorporated elemental Se, nor exhibited Se-dependent growth. However, 100% of Se-deficient mice (0.01 ppm Se), which express low levels of selenoproteins, succumbed to *F. tularensis*-live vaccine strain pulmonary challenge, whereas 50% of mice on Se-supplemented (0.4 ppm Se) and 25% of mice on Se-adequate (0.1 ppm Se) diet succumbed to infection. Median survival time for Se-deficient mice was 8 days post-infection while Se-supplemented and -adequate mice was 11.5 and >14 days post-infection, respectively. Se-deficient macrophages permitted significantly higher intracellular bacterial replication than Se-supplemented macrophages *ex vivo*, corroborating *in vivo* observations. Since Francisella replicates in alveolar macrophages during the acute phase of pneumonic infection, we hypothesized that macrophage-specific host selenoproteins may restrict replication and systemic spread of bacteria. *F. tularensis* infection led to an increased expression of several macrophage selenoproteins, suggesting their key role in limiting bacterial replication. Upon challenge with *F. tularensis*, mice lacking selenoproteins in macrophages (TrspM) displayed lower survival and increased bacterial burden in the lung and systemic tissues in comparison to WT littermate controls. Furthermore, macrophages from TrspM mice were unable to restrict bacterial replication *ex vivo* in comparison to macrophages from littermate controls. We herein describe a novel function of host macrophage-specific selenoproteins in restriction of intracellular bacterial replication. These data suggest that host selenoproteins may be considered as novel targets for modulating immune response to control a bacterial infection.

## Introduction

In both prokaryotes and eukaryotes the trace element selenium (Se) is co-translationally incorporated as selenocysteine (Sec) into selenoproteins (1, 2). Selenoproteins contain one or more Sec residues and have been shown to promote fitness of several bacterial pathogens (3). Bacterial selenoprotein enzymes including formate-dehydrogenase, xanthine dehydrogenase, hydrogenase-3 (FHL complex) (4), and glycine reductase, specific to *Clostridia*, promote the pathogen’s growth and fitness (5). Formate-dehydrogenase (Fdh), the most common selenoprotein expressed by bacteria (6), catalyzes the reversible two-electron oxidation of formate (7). Importantly, fdh is necessary for anaerobic metabolism of many bacteria (8) such as *Campylobacter jejuni*, a gut-dwelling bacterium and main causative agent of food borne illness worldwide (9). Mutant strains of *C. jejuni* deficient in formic acid metabolism exhibited reduced fitness and caused less severe enteric infection than the parent strain (10). Therefore, ablating the function of fdh results in loss of fitness, suggesting that selenoproteins are necessary for pathogen infectivity.

While selenoproteins promote normal growth, immunity, and reproductive health in humans (11), the ability of Se- supplementation and therefore, selenoproteins of the host to limit infectious disease severity has also been demonstrated in nutritional intervention studies (12–20). Se deficiency is associated with chronic infections caused by HIV/AIDS, hepatitis C virus, and *Mycobacterium tuberculosis* (12–15); nutritional interventions that contain Se have benefitted patients (16–20). However, many of these studies did not test the change in expression of selenoproteins following intervention. In addition, many of the nutritional Se supplementation studies were performed in Se-limiting geographical areas and likely had limited effect on the expression of selenoproteins. Sepsis is caused by a dysregulated immune response to infection, which usually originates from gram-negative bacteria resulting in life-threatening organ dysfunction, long term morbidity, and heightened risk of mortality (21). Plasma Se levels were lower in 92% of critically ill surgical patients than the standard value upon admission to the Intensive Care Unit (ICU). All patients exhibited decreased Se levels for the duration of the ICU stay, but strikingly, lower Se plasma concentrations were observed in patients with infection, tissue damage, organ dysfunction/failure and increased ICU mortality (22). Accordingly, several studies have noted a beneficial effect of Se supplementation as an adjunct therapy in patients with sepsis (23, 24). Se supplementation, and thus enhancement of selenoprotein function, may serve as a beneficial intervention to decrease infectious disease severity and improve patient outcome.

Previously, selenoproteins have been shown to be advantageous to the host by providing protection from inflammation and mediating resolution of infection as well as regulating overt immune responses (25–27). Selenoproteins are known to play a major role in maintaining cellular redox homeostasis and regulate a variety of biological processes such as intracellular calcium signaling (28, 29). The selenoprotein thioredoxin reductase 1 (TR1) is a pyridine nucleotide-disulfide oxioreductase that reduces disulfides to free thiols (30) and negatively regulates the HIV-1 encoded transcriptional activator, Tat, resulting in decreased HIV-1 replication in human macrophages (31). The action of TR1 in limiting viral replication, is one selenoprotein-dependent mechanism that may, in part, explain the therapeutic effects of Se supplementation in HIV/AIDS patients. Furthermore, macrophage-specific selenoproteins were found to be essential for clearance of the gastrointestinal nematode parasite, *Nippostrongylus brasiliensis* (27). An alternatively activated, or M2, macrophage response was deemed necessary for protection against helminthic infections, and Se supplementation of macrophages induced a phenotypic change from classically activated M1 to M2 (32). Macrophage selenoproteins were also necessary to control damaging proinflammatory responses in a mouse model of acute colitis, as Se-deficient and adequate mice exhibited increased colitis severity, inflammation and poor survival when compared to Se-supplemented mice (25). Additionally, in a model of gut inflammation induced by *Citrobacter rodentium*, a bacterium that induces murine enterohemorrhagic *Escherichia coli* (EHEC) infection, Se-deficient mice had increased mortality that was associated with poor integrity of colonic epithelial barrier cells in comparison to Se-adequate and supplemented mice (26). Although selenoproteins regulate many physiological processes under steady-state and inflammatory conditions, it is currently unknown if host selenoproteins are vital for antibacterial defense *via* limiting their intracellular replication.

In the current report, we utilize *F. tularensis* as a model organism to investigate the contribution of host vs. bacterial selenoproteins to the pathogenesis of pulmonary tularemia and disease outcome. *F. tularensis* is a facultative, intracellular, gram-negative bacterium that has broad host range (33, 34). Among the four subspecies of *F. tularensis*, *F. tularensis* spp. *tularensis* is most virulent to humans, followed by *F. tularensis* spp. *holartica*, *mediasiatica* and *F. novicida is considered more of an environmental organism that can cause disease in rodents* (34). Due to the lack of an approved vaccine, pathogen virulence, severity of pulmonary infection, ease of aerosolization, and its historical use as a bioweapon, *F. tularensis* ssp. *tularensis* is categorized as a Tier I select agent by the Centers for Disease Control (35, 36). Tularemia in humans is manifested in several forms owing to the route of infection that can cause cutaneous ulcers, glandular, ocular, typhoidal or pneumonic disease (37). The pneumonic form is an acute disease, resulting from the inhalation of as few as 10 colony-forming units (CFU) of *F. tularensis* and may result in a mortality rate of up to 60% if untreated (33, 38). Upon inhalation, *F. tularensis* preferentially infects phagocytes such as alveolar macrophages (39), and after several rounds of replication, spreads to tissue macrophages and dendritic cells in the lung, liver, spleen and lymph nodes (40–42). Additional cell types such as epithelial cells, hepatocytes, B cells, and red blood cells may support *F. tularensis* replication during the late stages of disease, but macrophages are the primary host cells during acute infection (39–42).

Bacterial incorporation of Sec into proteins involves the products of the *sel*A, *sel*B, *sel*C and *sel*D genes (43, 44). A unique tRNA specific for the UGA codon tRNA^(sec)^ that is encoded by *sel*C is initially charged with serine; the seryl moiety is then converted to a selenocysteinyl moiety (45). SelA encodes selenocysteine synthase that catalyzes the conversion of serine to selenocysteine, which requires selenophosphate, as a donor provided by selenophosphate synthetase that is encoded by *sel*D (46). Selenoprotein mRNA features a stem-loop secondary structure known as a SEleno Cysteine Insertion Sequence (SECIS) element and is located immediately downstream of a UGA codon in bacteria. However, in eukaryotes the SECIS element is located in the 3’untranslated region of the mRNA (28). For the UGA codon to be translated as Sec, a specialized translational elongation factor that is encoded by *sel*B must interact with the selenocysteine-tRNA^(sec)^, the SECIS element, and GTP at the ribosome (47). Thus, bacteria that translate proteins containing Sec generally require *sel*A, *sel*B, *sel*C, and *sel*D genes and their respective products. Alternatively, bacteria may utilize Se as a part of a unique 2-selenouridine (SeU) base in the wobble position of select tRNAs (48). Lastly, 2-selenouridine synthase (*YbbB)* can be synthesized independently of *sel*A, *sel*B, and *sel*C, but is thought to require *sel*D (48).

It was previously proposed that *F. tularensis* exploited host resources for the acquisition of Se for its fitness, as *F. tularensis holartica* Live Vaccine Strain (LVS) infection of A549 bronchial airway epithelial cells resulted in upregulation of host Se metabolism genes (49). However, in our current report, *in silico* sequence analysis determined that *F. tularensis* does not incorporate Se as a selenoprotein, a modified base, or as a cofactor. Additionally, *F. tularensis* does not incorporate elemental Se nor require Se for optimal *in vitro* growth, virulence gene expression, or *in vivo* infection. Se-adequate or supplemented mice exhibit greater survival from pulmonary tularemia, while Se-deficient mice succumb to *F. tularensis* LVS challenge. Bone-marrow derived macrophages (BMDMs) from dietary Se-adequate or Se-supplemented mice restricted bacterial replication in contrast to macrophages from Se-deficient mice. We then investigated whether the selenoproteins in macrophages were crucial in limiting the severity of pulmonary tularemia *in vivo*, as alveolar macrophages are the primary cells for *F. tularensis* replication. Transgenic mice that lack selenoproteins in macrophages (Trsp^M^) increasingly succumbed to pulmonary tularemia with a correspondingly greater bacterial burden in systemic tissues at later stages of infection. BMDMs from Trsp^M^ mice are unable to control *F. tularensis* LVS replication, a phenomenon that failed to be rescued by *ex vivo* Se supplementation, further demonstrating the importance of functional host macrophage selenoproteins in limiting bacterial replication. Herein, we provide a first report of a novel function of host macrophage selenoproteins in restriction of intracellular bacterial replication.

## Materials and Methods

### Mice

Four week-old C57BL/6 mice were maintained on purified diet (Tekland diets, Envigo, Madison, WI, USA) differing only in Na_2_SeO_3_ levels (deficient diet (TD.92163, <0.01 ppm Na_2_SeO_3_), adequate diet [TD.96363, 0.1 ppm (0.1 mg/kg) Na_2_SeO_3_], or supplemented diet [TD.07326, 0.4 ppm (0.4 mg/kg) Na_2_SeO_3_)] for greater than 12 weeks as previously described (27, 32).

TrspM mice lacking macrophage-specific selenoproteins were a kind gift from Dolph L. Hatfield at the Center for Cancer Research, National Institutes of Health, Bethesda, MD. TrspM mice are Trspfl/fl that are either heterozygous or homozygous for cre-recombinase under the lysozyme M promoter as previously described (50). TrspM and littermate control, WT mice, 6-8 weeks old, were maintained on standard chow diet that contains approximately 0.2 ppm of Se, which is also considered to be an ‘adequate’ level of Se diet. All animal experiments were conducted in accordance with Institutional Animal Use and Care Committee guidelines at the Pennsylvania State University.

### Bacterial Growth

Bacterial stocks were generated by expansion of 1 colony-forming unit (CFU) of *F. tularensis holartica* LVS in Chamberlain’s defined media (CDM) as previously described (51). Cultures were serially passaged at least 4 times in CDM under deficient [0nM sodium selenite (Na_2_SeO_3_ (Sigma- Aldrich, USA)], adequate (50nM Na_2_SeO_3_) or supplemented (200nM Na_2_SeO_3_) conditions. Bacterial growth assays were performed in 96 well flat bottom plates (Costar^®^, Corning^®^, Sigma- Aldrich, USA) with a starting OD600nM of *F. tularensis* LVS at 0.002 in CDM in the presence of 0nM, 50nM or 200nM Na_2_SeO_3_. OD600nM was recorded every 30 min for a period of 25 hr using a Spectromax spectrophotometer (Molecular Devices, CA, USA). Bacteria were enumerated by serial dilution in PBS (Hyclone™, GE Health Care, USA) and plated every 8 hr on chocolate agar plates prepared from Mueller-Hinton agar (Becton, Dickinson and Company, NJ, USA) supplemented with 1% (w/v) bovine hemoglobin (Reel™, ThermoFisher Scientific, USA) and 0.5% (v/v) IsoVitaleX™ (Becton, Dickinson and Company, NJ, USA). Plates were incubated at 37°C in an atmosphere of 5% CO2 for 72 hr before enumeration.

### *F. tularensis* Intranasal Infections

Mice were anesthetized with isofluorane and intranasally inoculated with 750, 1500 or 1750 CFU *F. tularensis* LVS in 50μl of PBS. Intranasal infections of mice with *F. tularensis* Schu S4 were performed under BSL3 conditions with Institutional Biosafety Committee approval at the Pennsylvania State University. CDC approved appropriate PPEs, procedures, and biosafety protocols were followed at the CDC certified ABSL-3 facility at PSU. Body weight was measured twice daily and mice were observed for changes in body condition. Mice were euthanized by CO2 asphyxiation at day 15 p.i. or if greater than 20% of initial body weight had been lost.

For bacterial enumeration, lung and spleen were homogenized in PBS with 1.0 mm Zircon/silicon beads (RPI Research products, IL, USA) in a Bead Blaster™24 (D2400, Benchmark Scientific, NJ, USA) using 6, 1 min cycles at a power level of 7 with a 30 sec break in between cycles. Whole livers were placed in whirl bags and crushed in 1mL of PBS. Lung, liver, spleen homogenates and blood were diluted and plated on chocolate agar (Becton, Dickinson and Company, NJ, USA). Plates were incubated at 37°C in an atmosphere of 5% CO2 for 72 hr before enumeration.

*In silico* analysis of genes required for the synthesis and incorporation of selenocysteine (Sec) or 2-selenouridine (SeU) Data was retrieved from the REFSEQ genomic and protein databases in FASTA file format. A blastp 2.5.0+ function using the default settings was performed on known selenoprotein sequences using the reference sequences from *E. coli* (NC_000913.3) and *D. vulargis* (NC_002937.3) against *F. tularensis* (NC_006570.2, NC_007880.1) sequences, and this was followed by a reciprocal function (52, 53). *F. tularensis* genomes were interrogated for tRNA, using the tRNADB-CE6 and GtRNAdb7 databases (54, 55). *E. coli* K12 was used a reference using the Conserved Domain Architecture Retrieval Tool (CDART) (56). Ontology was assessed using the Kyoto Encyclopedia of Genes and Genomes (KEGG) (57).

## Measurement of Bacterial Virulence Gene Levels by qPCR

Virulence gene expression was measured in bacteria grown to log phase in CDM with or without Se. RNA was extracted with TRIzol™ reagent (Invitrogen™, ThermoFisher Scientific, USA) and purified with an RNA isolation kit (Ambion™, ThermoFisher Scientific, USA). cDNA was prepared with an RT kit (BioRad, Hercules, CA, USA) and qPCR was performed with SYBR™ Green reaction mix (ThermoFisher Scientific, USA) using a CFX96 Touch™ Real-Time PCR Detection System (BioRad, Hercules, CA, USA). Fold change values were calculated by comparing the CT values of *fop*A(FTL _1328), *igl*C(FTL_1159 and *tul*4(FTL _0421) to the internal control *pol*A(FTL _1666). Primers (Integrated DNA Technologies, USA) are listed in **Supplementary Table 1**.

### Detection of Elemental Se by Atomic Absorption Spectrometry

*F. tularensis* LVS and *E. coli* (ssp. K-12) were grown in CDM or Brain Heart Infusion (BHI) (Becton, Dickinson and Company, NJ, USA) broth at 37°C overnight with agitation at 175 rpm in a final concentration of 0, 50 nM or 200 nM Na_2_SeO_3_. Cultures were washed thrice in PBS, centrifuging at 3000 × *g* for 20 min at 4°C. Pellets were then resuspended in 2 mL of MS grade water (Sigma-Aldrich, USA) and pulsed using a digital sonicator (Branson, Digital Sonifier^®^, Emerson, USA) for 3 cycles of 30 sec at an amplitude of 20. Bacterial lysates or control media were then filtered through a 0.45 μM filter and subjected to AAS to measure elemental Se by the Water Quality Laboratory, at the Penn State Institute of Energy and the Environment.

### Bone Marrow Derived Macrophages Isolation and Culture

BMDMs were prepared as described previously (58). In brief, the femur and tibia were crushed through a 70 μm strainer in complete DMEM (Hyclone™, SH3008102, GE Health Care, USA), 5% fetal bovine serum (Hyclone™, Lot# AWG18462, GE Health Care, USA tested to contain a very low level of Se- 25 μg/dl), 2mM L-Glutamine (Gemini Bio-Products, CA, USA), 1.5 mM HEPES (Corning, USA), 1 mM sodium pyruvate (Hyclone™, GE Health Care, USA) and 1X nonessential amino acids (Hyclone™, GE Health Care, USA). Cells were centrifuged at 400 × *g* for 10 min at 25°C, the supernatant was decanted and pellet resuspended in complete DMEM with 20% (v/v) L929 conditioned media that was generated using low Se FBS as described above [containing macrophage-colony stimulating factor (M-CSF)]. Bone-marrow progenitor cells were plated at a density of 5 × 105 cells/100cm2 petri dish (VWR, USA) in deficient (0 nM), adequate (50 nM Na_2_SeO_3_) or supplemented (200 nM Na_2_SeO_3_) culture conditions. On days 3 and 5 of culture, media was aspirated and replenished with complete DMEM media containing 20% L929 with or without Na_2_SeO_3_. On day 7 of culture, BMDMs were harvested and utilized to assess Se status of diet mice by measurement of GPX1 expression or were allocated for gentamicin protection assays.

### Gentamicin Protection Assays

BMDMs were seeded at a density of 3-5 × 105 cells/well and infected at an MOI of 1:100 CFU in a 24 well tissue culture treated plate (CellStar^®^, VWR, USA). Cells were centrifuged at 300 x *g* at RT for 10 min and then incubated at 37°C, 5% CO2 for 20 min. Media was aspirated, fresh DMEM complete media containing gentamicin (100 μg/mL) (Gibco™, ThermoFisher Scientific, USA) was added and cells were incubated at 37°C, 5% CO2 for 1 hr to remove extracellular bacteria. BMDMs were washed thrice with PBS and lysed with a solution of 0.1% deoxycholate in PBS solution for 2-5 min at RT for CFU enumeration at 2hr post-inoculation. Remaining cells were resuspended in DMEM complete media and lysed 18 hr later. Cell lysates from both time points were serially diluted in PBS and plated for bacterial enumeration.

### RNA-Sequencing and Differential Expression Analysis

Three biological replicates of infected and uninfected BMDMs from gentamicin protection assays at 10hr p.i. were collected and processed for RNA isolation using a combination method incorporating both TRIzol reagent and a Purelink™ RNA Mini Kit (Invitrogen, Carlsbad, CA). Transcriptome library preparation and subsequent sequencing was done through BGI Genomics (Cambridge, MA) using their unique DNBseq platform. 100bp paired end reads were obtained at an average coverage of 35.2 million aligned reads/sample. We used FastQC for quality control assessment and STAR v. 2.7.3a (59) to align the raw sequencing reads to GRCm38.p6 using gene coordinates from the GENCODE database Release M25 (60). Duplicate reads were tagged using Picard v. 2.9.0 (61). Mapping quality was checked with transcript integrity scores using RSeQC v. 3.0.1 (62), and genes were quantified for expression using RNA-SeQC v. 2.3.5 (63). We then checked for clustering of samples with multidimensional scaling analysis and corrected for batch effects using Combat-Seq (64). Principle component analysis was performed, and batch 3 samples were removed due to poor clustering with other replicates. Genes were filtered for sufficient coverage using the edgeR function filterByExpr to filter out genes that had less than 10 reads in more than half of the samples (65). We conducted differential expression analysis on infected vs. uninfected macrophages using edgeR v. 3.30.0 (65) RNA-seq data can be accessed at BioSample accession SAMN19317215

### Statistical Analyses

Log-Rank Mantle-Cox tests analyzed survival data from *in vivo F. tularensis* LVS challenge in Se diet and Trsp^M^ mice. One-way ANOVA with Tukey’s *post hoc* test analyzed data from gentamycin protection assays and Bonferroni’s multiple comparison test was used to analyze relative GPX1 expression in BMDMs. Two-way ANOVA with Sidak’s *post hoc* test analyzed data from *in vivo* bacterial burden enumeration and gentamycin protection assays from BMDMs of Trsp^M^ mice. All statistical analyses were performed using Prism (version 5) (GraphPad Software, San Diego, CA, USA).

## Results

### *F. tularensis* Does Not Possess the Genes Necessary for Sec Biosynthesis or Se Incorporation

It was previously reported that *F. tularensis* LVS upregulates expression of genes associated with Se-metabolism in alveolar epithelial cells. These findings led to speculation that *F. tularensis* may sequester Se for some critical biological processes (49). We therefore investigated whether *F. tularensis* requires Se for any specific metabolic processes. To determine if *F. tularensis* can incorporate Sec into proteins, we probed *F. tularensis* genomes using bioinformatics tools and databases. First, a blastp2.5.0+ tool (66) was used to identify putative selenoproteins in the *F. tularensis* proteome. The use of protein sequences overcame an inherent codon usage bias between reference organisms and *F. tularensis*. The protein blastp function was enhanced by the curated domain (CD) search tool, but resulted in hits with extremely low scores and high E-values (67). Even though the search identified a potential tRNA (*selC*) and formate dehydrogenase (*fdh*) in *F. tularensis*, the identity was exceedingly low suggesting that the bacterium may not be able to synthesize selenocysteine.

Next, we investigated whether *F. tularensis* encodes tRNA^(sec)^ using tRNADB-CE6 and GtRNAdb7 databases (54, 55). This confirmed the presence of tRNA^(sec)^ in known Sec incorporating bacteria (*E. coli* and *D. vulgaris*), but failed to identify the tRNA^(sec)^ in any published *F. tularensis* genomes, including LVS and Schu S4 (**Table 1**). The lack of a tRNA^(sec)^ with the appropriate anticodon strongly suggests that *F. tularensis* is unable to incorporate Sec into proteins, and therefore do not express any functional selenoproteins.

**Table 1:** Genes required for synthesis and incorporation of selenocysteine in reference bacteria and genomes of bacteria were probed for expression of Sec biosynthesis genes and alternatively, selenouridine (SeU) synthesis, which are two of the three known mechanisms of Se utilization in prokaryotes.

Gene	Product	Function	*E. coli*	*D.vulgaris*	*F.t* ssp. *Holartica* LVS	*F.t* ssp. *tularensis* Schu S4
***selA***	Selenocysteine synthase	generates selenocysteine from serine + selenophosphate	**Present**	Present	**Absent**	Absent
***selB***	selB	elongation factor for selenocysteine	**Present**	Present	**Absent**	Absent
***selC***	tRNA^Sec^	SeCys tRNA, specific for UGA codons	**Present**	Present	**Absent**	Absent
***selD***	Selenophosphate synthetase	generates selenophosphate, the Se donor required by selenocysteine synthase	**Present**	Present	**Absent**	Absent
***ybbB***	tRNA 2- selenouridine synthase	catalyzes 2- thiouridine to 2- selenouridine from selenophosphate donor	**Present**	Absent	**Absent**	Absent

To determine whether other components of the Sec incorporation machinery are present in *F. tularensis*, we utilized CDART and KEGG databases (56, 57). Using the amino acid sequences of *E. coli sel*A and *sel*B gene products as templates, CDART identified architectures comprised of two and five conserved domains, respectively. Filtering by the NCBI taxonomy tree revealed that these domain architectures were found in the genomes of other known Sec incorporating bacteria, *e.g., D. vulgaris*, but were absent in all *F. tularensis* genomes. Furthermore, individual domains specific to selenometabolism, *e.g*., Sec synthase N-terminal domain in *sel*A and *sel*B-winged helix domain in *sel*B, were completely absent in *F. tularensis*, suggesting that functional copies of these genes are not present. KEGG resources also demonstrated that in all available *F. tularensis* genomes, *sel*B was not among the translation elongation factors. Additionally, *sel*A, *sel*D, and SeU were absent in the KEGG selenocompounds metabolism pathway in *F. tularensis*, while they were all present in *E. coli* and *D. vulgaris* (excluding *Ybb*B). *In silico* analyses of genes necessary for Sec incorporation (*selA-D*) or an alternative mechanism of Se utilization (*YbbB*) revealed that *F. tularensis* ssp. *holartica* LVS and *F. tularensis* ssp. *tularensis* Schu S4 lacked these genes, while *E. coli* and *D. vulgaris* possess the genes vital for Se utilization (**Table 1**).

### Se Supplementation Does Not Alter *F. tularensis* LVS Growth, *Ex Vivo* Entry and Replication In Host Cells, or Lung Colonization

To validate results from bioinformatics analyses, *F. tularensis* LVS was cultured in the absence or presence of Se to examine growth kinetics, cellular entry, intracellular replication, and *in vivo* infectivity. *F. tularensis* LVS cultures containing Se did not display altered growth kinetics in comparison to cultures deficient in Se as measured by optical density (**Supplementary Figure 2A**) and CFU over a period of time (**Supplementary Figure 2B**). Furthermore, expression of key virulence genes of *F. tularensis* LVS were not perturbed by addition of Se to liquid culture (**Supplementary Table 2**). Since macrophages are a primary site of *F. tularensis* replication during disease pathogenesis (39), we next asked if Se addition *ex vivo* could influence *F. tularensis* LVS entry or intracellular replication. BMDMs from Se-deficient mice were inoculated with *F. tularensis* LVS in the absence or presence of Se. Bacterial entry at 2 hr post-inoculation (**Supplementary Figure 3A**) and burden at 24 hr post-inoculation (**Supplementary Figure 3B**) were found to be identical between Se-deficient and supplemented conditions, indicating *in vitro* Se supplementation does not affect bacterial entry or replication. Additionally, Se-deficient mice were intranasally inoculated with *F. tularensis* LVS grown in Se-deficient or supplemented conditions to measure the ability of Se to regulate *in vivo* bacterial colonization and replication. Pulmonary bacterial burden was comparable at 6, 12, and 24 hr post-inoculation between Se-deficient and supplemented mice (**Supplementary Figure 4**). These data indicate that Se does not alter the physiology or infectivity of the pathogen, *F. tularensis.*

### *F. tularensis* LVS Does Not Accumulate Elemental Se

**Figure 1:**
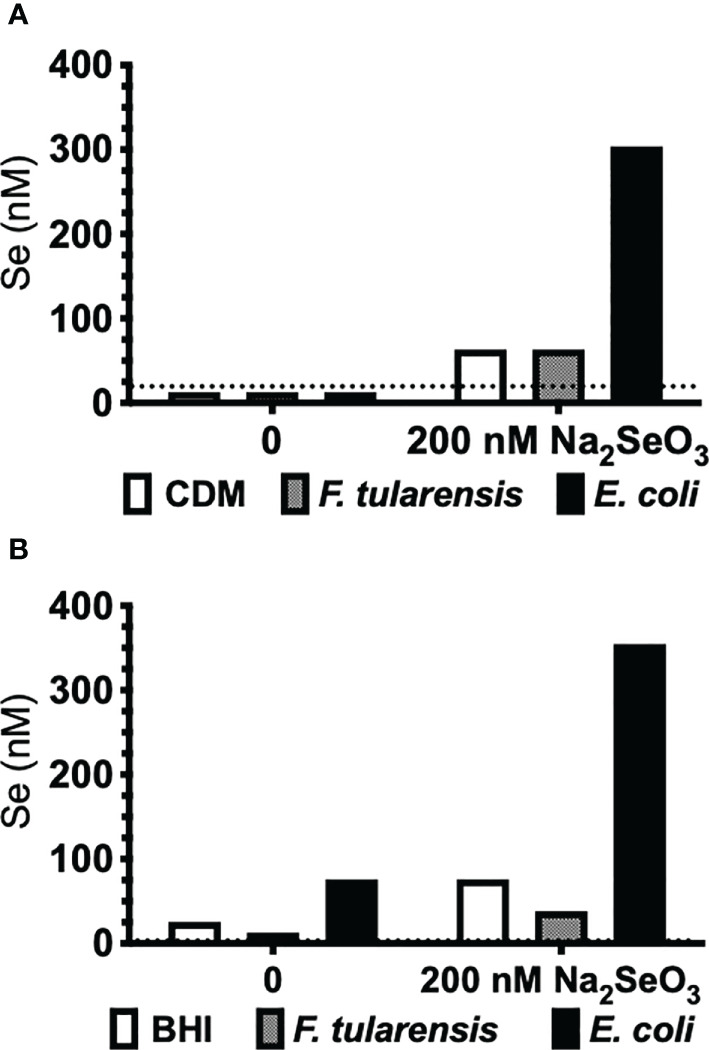
*F*. *tularensis* LVS does not incorporate Se. *F*. *tularensis* LVS or *E*. *coli* K12 was grown to saturation in **(A)** CDM or **(B)** undefined BHI broth in the presence (200 nM) or absence (0 nM) of Na_2_SeO_3_. Elemental Se concentration in bacterial lysates was measured by AAS. Data are representative of three independent experiments.

The third known mechanism of Se utilization in prokaryotes, Se incorporation as a cofactor in molybdenum hydroxylases (68), could not be examined by bioinformatics analyses. *Francisella* primarily utilizes copper and zinc as cofactors, but not Se as predicted by a protein database of trace element utilization (69). Nonetheless, we further confirmed that *F. tularensis* does not accumulate Se by this third mechanism of utilization by measuring incorporation of elemental Se. *F. tularensis* LVS and *E. coli* were grown to stationary phase under deficient (0 nM) or supplemented (200 nM Na_2_SeO_3_) conditions in defined Chamberlain’s defined media (CDM) or undefined brain-heart infusion (BHI) media, and bacterial lysates were subjected to AAS (atomic absorption spectroscopy) analysis. Indeed, Se was not incorporated into *F. tularensis* LVS as lysates yielded, at most, only the concentration of Se originally added to culture (**Figure 1**). However, *E. coli* had an appreciable concentration of elemental Se when cultured in Se-supplemented CDM (**Figure 1A**) as well as detectable levels in BHI (**Figure 1B**) and increased levels in Se-supplemented BHI. Taken together, *in silico*, gene level, growth kinetics, and AAS analyses determined that *Francisella* does not incorporate Se as a selenoprotein, as a modified base, or as a cofactor.

### Dietary Se Deficiency Leads to Increased Susceptibility to Pulmonary Tularemia

**Figure 2:**
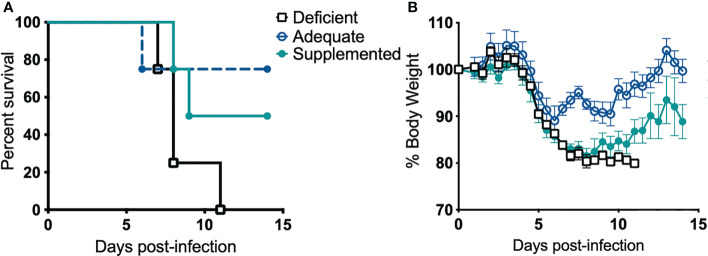
Dietary Se is required for survival from *F*. *tularensis* LVS challenge. Se-deficient, -adequate or -supplemented diet mice (n= 4/group) were intranasally inoculated with 1500 CFU of *F*. *tularensis* LVS and **(A)** survival and **(B)** body weight was monitored daily for 14 days. Statistical significance of survival was assessed using the Log-rank (Mantel-Cox) test. Daily weights represent the mean % body weight of the surviving animals; error bars denote +SEM. Data are representative of three independent *in vivo* experiments with similar outcomes.

Next, we examined if Se influences disease pathogenesis. Alteration of dietary Se is a well-established model to change Se status and thus selenoprotein expression and function in mice (25–27, 32, 70). Mice maintained on Se-deficient, -adequate, or -supplemented purified diets for at least 12 weeks were intranasally infected with *F. tularensis* LVS, and body weight and survival were monitored for 14 days post-infection (p.i.). All deficient animals succumbed to infection with 1500 CFU by day 11 p.i. (**Figure 2A**). In contrast, adequate and supplemented mice exhibited increased survival rates (75% and 50%, respectively) and median survival for Se-deficient mice was day 8 p.i. while Se-supplemented mice was day 11.5 p.i. (**Figure 2A**). There was no statistical difference in the rate of survival between Se-supplemented and Se-adequate mice. Consistent with survival, Se-adequate and supplemented mice displayed decreased weight loss in comparison to surviving Se-deficient mice (**Figure 2B**), suggesting that Se status of the host influences *F. tularensis* LVS infection severity.

### Se-Supplementation of Macrophages Limits *F. tularensis* LVS Replication

**Figure 3:**
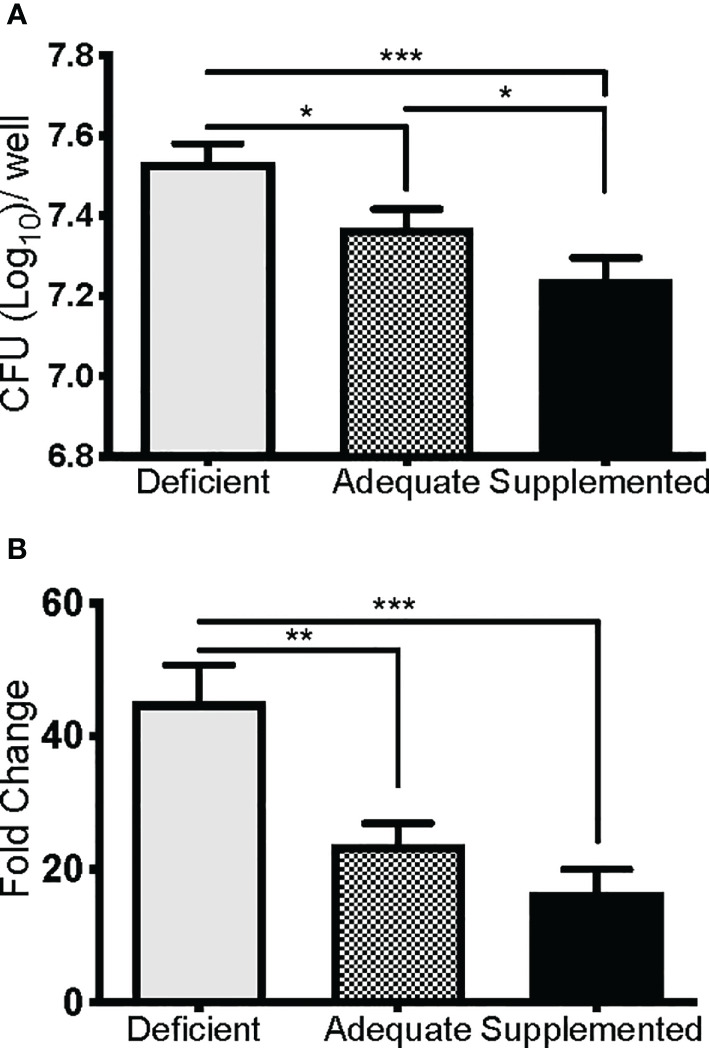
Se restricts *F*. *tularensis* LVS replication in macrophages. BMDMs from Se-deficient, -adequate, or -supplemented mice were maintained *ex vivo* under Se deficient (0 nM), adequate (50 nM) or supplemented (200 nM) conditions (n=3/one biological replicate, n=3 biological replicates/diet group), infected with *F*. *t* LVS at an MOI of 1:100. **(A)** Intracellular growth of bacteria was enumerated at 24 hr post-infection. **(B)** Bacterial growth over 24 hr represented as fold change in bacteria enumerated at 2 hr post infection. Data are depicted as the mean of three biological replicates and error bars denote +SD. Statistical significance was assessed by One-way ANOVA with Tukey’s Multiple Comparison Test (*p< 0.05) and data are representative of three independent experiments. **p<0.01, ***p<0.001.

We next determined if the observed lethality of Se-deficient mice from pulmonary tularemia was due to an inability to control bacterial replication and systemic dissemination. Since macrophages are the initial site of *F. tularensis* infection and replication (39), and Se is known to regulate macrophage function *via* selenoproteins (32, 71, 72), we hypothesized that this compartment of the immune system was compromised under Se-deficient conditions, leading to an inability to control bacterial replication. Se status of mice was first confirmed by measuring expression of the selenoprotein GPX1 in BMDMs, which was significantly greater as dietary Na_2_SeO_3_ concentration increased (Deficient vs. Adequate, ***p<0.001; Adequate vs. Supplemented ***p<0.001) (**Supplementary Figure 5**). To measure intracellular growth of *F. tularensis* LVS, a gentamicin protection assay was performed with BMDMs cultured from Se diet mice and maintained under respective deficient, adequate, or supplemented conditions *ex vivo*. Se-deficient macrophages had the greatest bacterial burden 24 hr p.i. compared to Se-adequate (*p<0.05) and supplemented macrophages (***p<0.001, **Figure 3A**). Interestingly, when the growth of bacteria was measured by normalizing CFU at 24 hr to 2 hr p.i., Se supplementation limited bacterial growth in a dose-dependent manner as less growth was observed in adequate (**p<0.01) and supplemented (***p<0.001) groups in comparison to deficient (**Figure 3B**). Therefore, the presence of Se restricts *F. tularensis* LVS intracellular growth in macrophages, which may provide an advantage to the Se-adequate and supplemented hosts during pulmonary challenge.

We then determined if BMDMs supplemented with alternative selenocompounds would have similar GPX1 expression patterns, and if these compounds could also limit intracellular bacterial growth. BMDMs were supplemented with 200 nM of methylseleninic acid (MSA), selenomethionine (SeMet), Na_2_SeO_3_, or maintained under deficient conditions for 24 hr. BMDMs supplemented with MSA and Na_2_SeO_3_ had reduced intracellular growth compared to BMDMs maintained in deficient media or supplemented with SeMet (**Supplementary Figure 6A**). Additionally, GPX1 protein expression levels in MSA and Na_2_SeO_3_ treated BMDMs were higher than levels of deficient or SeMet treated BMDMs (**Supplementary Figure 6B**); thus, supporting the hypothesis that bacterial replication restriction was influenced by selenoprotein expression levels. We speculate that the supplementation of SeMet in BMDMs did not result in an increase of selenoproteins as macrophages do not express methioninase-γ-lyase (72), an enzyme required for the utilization of SeMet in the methylselenol pool (73). In summary, Se status of the host and *ex vivo* supplementation clearly limited bacterial growth and replication in macrophages, while suggesting an important role for host selenoproteins in the process.

### Macrophage-Specific Selenoproteins Are Essential for Survival From Pulmonary Tularemia

**Figure 4:**
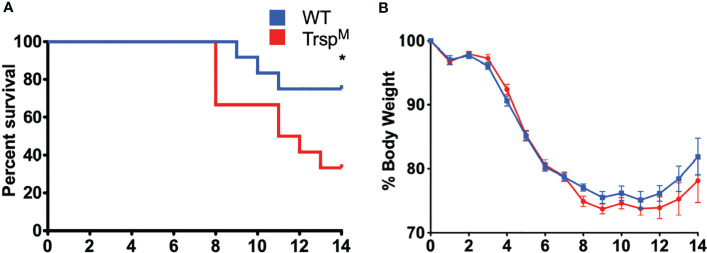
Macrophage selenoproteins are required for survival from *F*. *tularensis* LVS infection. Six- to eight-week-old WT and Trsp^M^ mice (n= 8-16/group) were inoculated with 750 CFU of *F*. *tularensis* LVS and **(A)** survival and **(B)** body weight were monitored for 14 days. Mice that lost greater than 20% of body weight were euthanized. Statistical significance of survival was assessed by the Log-rank (Mantel-Cox) test (*p<0.05). Weights were represented as the mean % body weight of the surviving animals. Error bars denote +/-SEM and data are representative of three independent experiments.

Selenoproteins in macrophages are necessary for beneficial immune responses (26, 27). As described above, Se-deficient animals succumbed to *F. tularensis* LVS intranasal challenge (**Figure 2A**), Se-deficient BMDMs were more permissible to bacterial replication (**Figure 3B**), and intracellular growth of bacteria was inversely related to the level of macrophage selenoproteins (**Supplementary Figure 6A**). We therefore sought to determine the contribution of macrophage-specific selenoproteins during pulmonary tularemia in mice that lack selenoproteins in macrophages (Trsp^M^), as previously described (44). The selenocysteinyl tRNA (Trsp^fl/fl^) gene was disrupted using Cre/loxP system when co-expressed with Cre recombinase under the lysozyme M promoter. Mature macrophages express lysozyme M, thus resulting in a disruption of the Trsp gene product and an inability to synthesize selenoproteins. We confirmed the deletion of selenoproteins in macrophages of Trsp^M^ mice by measuring GPX1 expression in BMDMs derived from WT and Trsp^M^ mice. Indeed, GPX1 expression is absent in Trsp^M^ macrophages (**Supplementary Figure 7**). Trsp^M^ and WT littermate controls were intranasally infected with 750 CFU of *F. tularensis* LVS. Trsp^M^ mice succumbed to infection more quickly than WT mice (**Figure 4A**) with concordant weight loss (**Figure 4B**), suggesting that macrophage-specific selenoproteins are protective against pulmonary tularemia. The LD100 for Trsp^M^ mice was 1500 CFU (**Supplementary Figures 8A, B**) and 1750 CFU for WT mice (**Supplementary Figures 8C, D**), indicating that the presence of functional selenoproteins cannot protect mice from pulmonary tularemia at higher inoculating doses.

### Macrophage-Specific Selenoproteins Limit Bacterial Replication in Systemic Tissues

**Figure 5:**
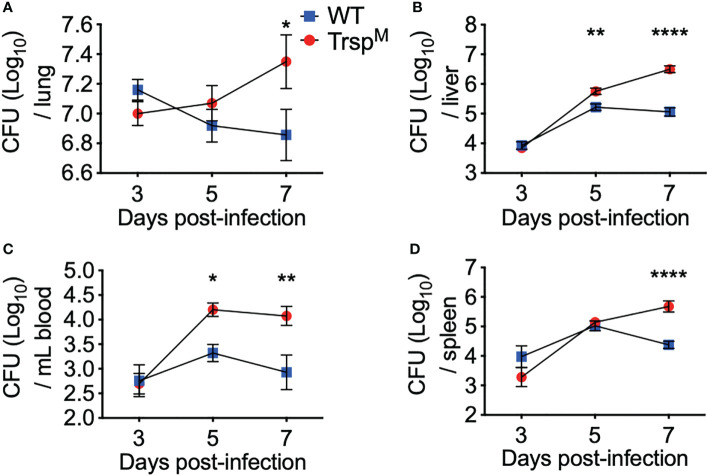
Macrophage selenoproteins are required for control of *F*. *tularensis* LVS infection in mice. WT (blue square) and Trsp^M^ (red circle) mice were intranasally inoculated with 750 CFU of *F*. *tularensis* LVS and at day 3, 5 or 7 p.i. mice were euthanized and bacterial burden was enumerated from the **(A)** lungs, **(B)** liver, **(C)** blood and **(D)** spleen. Statistical significance was assessed by two-way ANOVA with Sidak’s *post-hoc* test (*p<0.05). Data are representative of the mean and error bars +/-SEM. (ABD) Day 3 data were combined from three separate *in vivo* experiments (n=9/genotype), and four separate *in vivo* experiments at day 5 and 7 (n=14/genotype). **(C)** Day 3 data were combined from two separate *in vivo* experiments (n=6/ genotype) and day 5 and 7 data were combined from three separate *in vivo* experiments (n=11/ genotype). **p<0.01, ****p<0.0001.

To further elucidate the protective contribution of macrophage-specific selenoproteins, Trsp^M^ and WT littermate controls were challenged with *F. tularensis* LVS, and bacterial burden was assessed in the lung, liver, blood, and spleen throughout infection. Although no differences were observed at day 3 p.i., bacterial burden was increased in Trsp^M^ mice in the liver and blood at day 5 p.i. (**Figure 5B** **p<0.01, 5C *p<0.05) and in the lung and the spleen at day 7 p.i. (**Figure 5A** *p<0.05, 5D ****p<0.0001) in comparison to WT controls. Presence of macrophage-specific selenoproteins in littermate controls also limited bacterial replication in the liver and blood on day 7 (**Figure 5B** ****p<0.0001, 5C **p<0.01) in comparison to Trsp^M^ mice. These data confirm that macrophage-specific selenoproteins limit bacterial replication at both the site of infection and in systemic tissues at later stages of the infection, and thus, promote survival from pulmonary tularemia challenge (**Figure 4A**).

### Macrophage Selenoproteins Limit Intracellular *F. tularensis* LVS Replication

**Figure 6:**
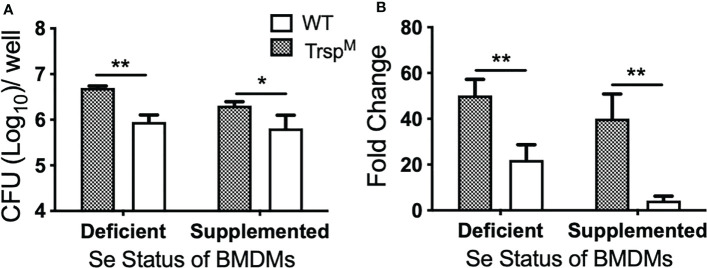
Macrophage selenoproteins restrict intracellular replication of *F*. *tularensis* LVS. BMDMs from WT and Trsp^M^ mice (n=3/group) were infected with *F*. *tularensis* LVS at an MOI of 1:50 and intracellular bacteria were enumerated at 24 hr p.i. **(A)** The number of bacteria recovered at 24 hr. Line represents the mean and error bars denote +SD. **(B)** Bacterial growth over 24 hr represented as fold change. Statistical significance was assessed by one-way ANOVA followed by Bonferroni’s multiple comparison test (*p<0.05). Data are representative of three experiments. **p<0.01.

The role of selenoproteins in direct antibacterial defense is poorly understood. Since macrophage selenoproteins limit the severity of *F. tularensis* infection (**Figures 4**, **5**), it was necessary to establish if bacterial replication restriction observed in the Se dietary model (**Figure 3B**) was mediated in a selenoprotein-specific manner. Indeed, BMDMs from Trsp^M^ mice were more permissive to *F. tularensis* replication in comparison to WT littermate controls as measured by CFU (* p<0.05, **Figures 6A**) and fold change replication at 24 hr over 2 hr (**p<0.01, **Figure 6B**). Moreover, the failure to limit replication that was observed in Trsp^M^ macrophages could not be rescued by the addition of Na_2_SeO_3_ (**p<0.01 Trsp^M^ vs. WT, **Figure 6B**), indicating that replication restriction is strictly by a selenoprotein-mediated mechanism. Experiments with *F. tularensis* Schu S4 yielded similar results (data not shown) indicating that macrophage specific selenoproteins play a role in limiting the replication of virulent *F. tularensis*.

### Infected WT Macrophages Show Increased Expression of Six Selenoproteins

**Figure 7:**
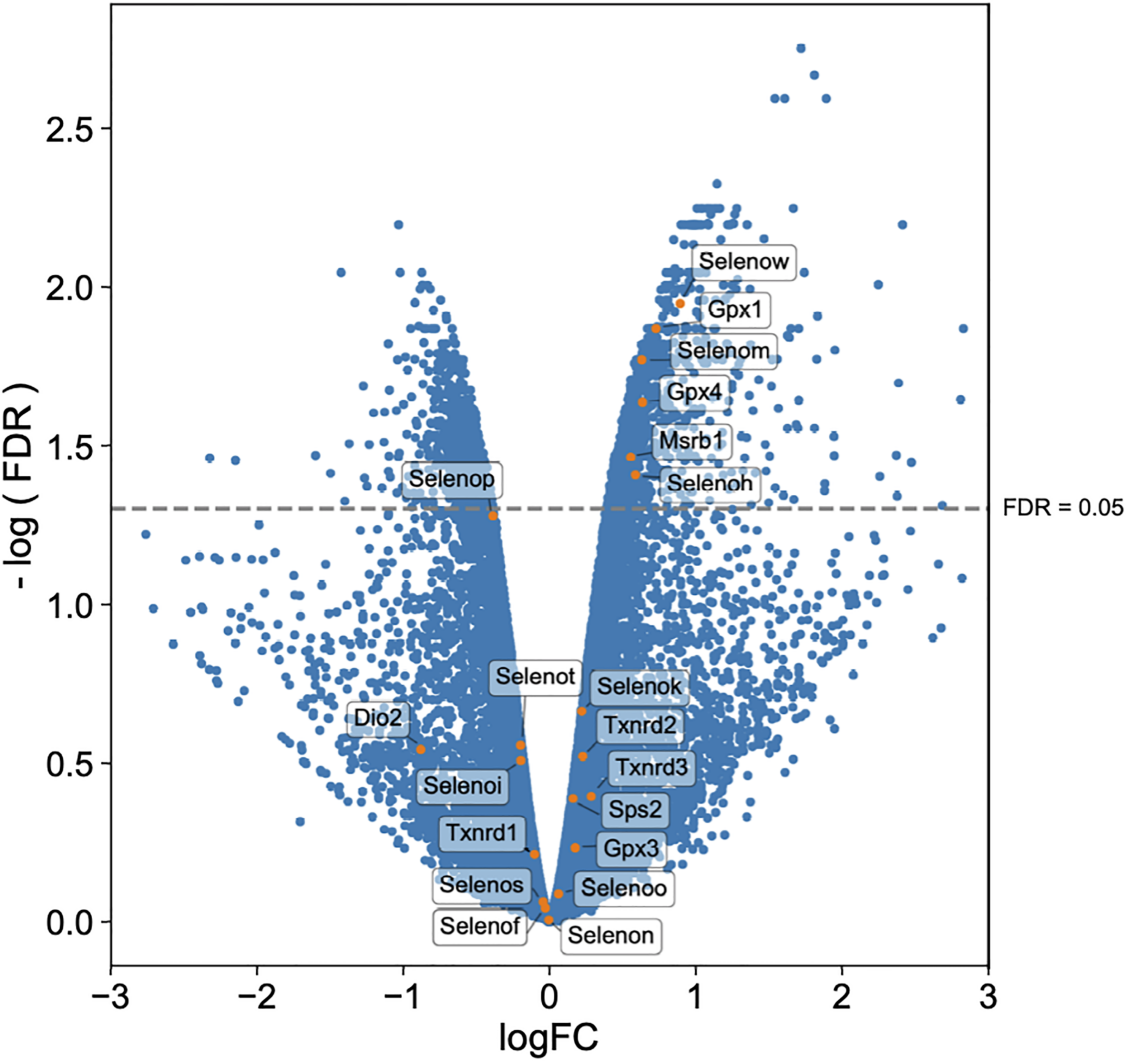
Selenoproteins are differentially regulated between infected and uninfected macrophages. BMDMs from WT mice (n=3/group) were infected with *F. tularensis* LVS at an MOI of 1:10. Cells were harvested at 10hr p.i., and processed for RNA isolation and subsequent sequencing. Volcano plot of differentially expressed genes shows that 20 of 24 murine selenoproteins are differentially expressed when comparing infected BMDMs to uninfected controls, with six selenoproteins showing significance. Differential expression analysis was conducted using edgeR v. 3.30.0 (FDR <0.05).

Twenty-five and 24 unique selenoproteins have been identified in the human and murine genomes, respectively (28). Many of the selenoproteins have been classified according to their function into separate groups associated with redox signaling, protein folding, antioxidative capacity, and others. There are also several selenoproteins whose functions are as yet unknown (29, 74). To more clearly elucidate which selenoproteins might be important for the restriction of bacterial replication in macrophages, we performed transcriptomic analyses. Gentamicin protection assays were performed with BMDMs isolated from WT mice and infected with *F. tularensis* LVS. At 10hr p.i., both infected and uninfected cells were harvested and processed for RNA isolation and subsequent sequencing. RNA-seq results revealed 15,393 genes with sufficient coverage, and 1,866 genes that were differentially expressed between the infected and uninfected groups with a false discovery rate (FDR) of less than 0.05. Of the significant differentially expressed genes, six were confirmedto be selenoproteins (**Figure 7**). mRNA expression levels of Selenoprotein W (SelenoW), Glutathione peroxidase1 (Gpx1), SelenoM, Gpx4, Methionine sulfoxide reductase B (Msrb1), and SelenoH were all elevated in infected macrophages when compared to uninfected controls. Although not significant, an additional 14 selenoproteins were found to be differentially expressed between the groups (**Figure 7**, **Supplementary Table 3**). SelenoW, which has been suggested to have antioxidant functions as well as a potential for mediating cellular immunity (75, 76), was one of the differentially expressed selenoprotein at the RNA level. This observation was confirmed *via* qPCR analysis of the same infected and uninfected BMDM groups (**Supplementary Figure 9**). However, protein expression patterns was not significantly different between uninfected and infected cells as determined by western blot analysis in two representative selenoproteins, Gpx1 and Gpx4 (**Supplementary Figure 10**). Similarly, no significant difference between protein levels of SelenoW, SeleneoM, and MsrB1was observed (data not shown).

## Discussion

Several studies have utilized dietary animal models fed with various levels of Se to investigate the role of Se in infectious diseases. In a model of central nervous system listeriosis induced by the intracellular bacterium, *Listeria monocytogenes*, Se-deficiency led to greater central nervous system lesion development (77). In a separate murine model of listeriosis, Se-deficient mice had impaired innate immune cell responses such as decreased splenic NK cell cytotoxicity and serum proinflammatory cytokine (IL-12) production and consequently exhibited increased bacterial burden in the liver, spleen and brain in comparison to Se-adequate mice (78). Additionally, Se deficiency during *Staphylococcus aureus*-induced mastitis exacerbated the proinflammatory cytokine response due to suppressed PPARγ activity, and increased NF-κB activation, that resulted in increased nitric oxide levels and larger inflammatory lesions (79). In a model of bacterial induced gut inflammation, Se-deficient mice challenged with *C. rodentium* had decreased group 3 innate lymphoid cells and T helper 17 cells in comparison to mice with adequate or supplemented Se-status. This altered immune response at the site of infection was associated with decreased epithelial integrity and increased mortality of Se-deficient mice (26). We observed increased severity of disease in Se-deficient mice in response to pneumonic challenge of *F. tularensis* (**Figure 2**). Our findings are in agreement with previous studies suggesting that adequate dietary Se-status of the host is necessary for proper immune function and resolution of bacterial infection. In addition, for the first time our studies demonstrate Se- and selenoprotein-dependent restriction of bacterial replication in macrophages (**Figure 3**).

Transcriptome analysis of a bronchial airway epithelial cell line during *F. tularensis* LVS infection revealed upregulation of Se metabolism genes highlighting the importance of Se utilization traits. It was proposed that *F. tularensis* utilizes this strategy to exploit host resources for pathogen fitness (49). Surprisingly, bioinformatics (**Table 1**) and AAS analyses (**Figure 1**) revealed the inability of *F. tularensis* LVS to express Se utilization traits or incorporate Se into its proteome. We therefore hypothesized that the upregulation of genes associated with selenoamino acid incorporation, may be a protective mechanism employed by the host to limit bacterial replication. We then determined that Se status of the host impacts the severity of pulmonary tularemia, as Se-deficient mice succumbed to *F. tularensis* challenge, while animals of adequate and supplemented Se status exhibited greater survival and decreased weight loss (**Figure 2**). Macrophages are the initial cellular target for *F. tularensis* infection, replication, and expansion during the pathogenesis of tularemia (39). We demonstrated that BMDMs of adequate or supplemented Se status limited bacterial replication in comparison to Se-deficient BMDMs (**Figure 3**). As Se status of mice increased, so did selenoprotein expression in BMDMs (**Supplementary Figure 5**), providing correlative evidence that host selenoproteins may function to limit intracellular replication. *F. tularensis* challenge of Trsp^M^ mice revealed that macrophage-specific selenoproteins are necessary for survival from pulmonary tularemia as Trsp^M^ mice succumbed to the disease, whilst WT littermate controls survived (**Figure 4**). Greater bacterial burden in systemic tissues such as the spleen and liver later during infection in Trsp^M^ mice (**Figure 5**) suggested that absence of macrophage selenoproteins leaves the host unable to control *F. tularensis* replication. Indeed, BMDMs from Trsp^M^ mice permitted greater bacterial replication in comparison to WT littermate controls (**Figure 6**), thus demonstrating the necessity of macrophage-specific selenoproteins in limiting bacterial replication. Although bactericidal activities in macrophages have been demonstrated to be enhanced by Se-supplementation (80, 81), no studies have thus far demonstrated a specific role for macrophage-specific selenoproteins in bacterial growth.

Interestingly, RNA expression of six selenoproteins Gpx1, Gpx4, Msrb1, SelenoH, SelenoM, and SelenoW, were upregulated during *F. tularensis* infection of WT BMDMs (**Figure 7**). Gpx1 and Gpx4 have well-characterized antioxidant functions, reside in the cytoplasm and mitochondrial membrane and are ubiquitously expressed throughout the body (82). MsrB1 catalyzes reversible stereoselective methionine oxidation of the *R* enantiomer of oxidized methionine residues in proteins (83). As MsrB1 function decreases during Se-deficiency, innate immunity is compromised in macrophages owing to the disruption of actin polymerization-dependent processes, *e.g*., filopodia formation, micropinocytosis (84), and cytokine release (85). SelenoM resides in the ER membrane and although its function is unknown, it may be involved in neurodegeneration as it is most highly expressed brain tissue (86). Both SelenoH and SelenoW possess antioxidant functions, belong to the thioredoxin-like family of selenoproteins (76). SelenoW is expressed during the development of the nervous system, skeletal muscles and heart and may protect developing myoblasts from oxidative stress (87), but also mediates cell immunity (88). Contribution of these selenoproteins to antibacterial defense in macrophages is not known. Interestingly, mRNA expression of the selenoprotein genes *gPx1*, *msrB1*, *selenoW* and *selenoH* are most affected by Se availability and translation is reduced during Se-deficiency (89). This group of selenoproteins are therefore referred to as ‘stress selenoproteins’ (29) and *F. tularensis* infection may be inducing Se-deficiency in host cells as Se is utilized *via* selenoproteins to limit bacterial replication. Alternatively, *F. tularensis* infection leads to macrophage stress, which in turn may lead to upregulation of these selenoproteins. However, we did not observe a statistically significant increase in the expression of these selenoproteins at the protein level (**Supplementary Figure 10**). This may be due to the limited availability of Se in the medium due to perhaps increased utilization of Se by infected stressed cells, delayed and hierarchal expression kinetics of selenoproteins based on the availability of Se, and delayed translation of selenoproteins. Therefore, further in-depth studies are needed to establish the expression pattern and specific role of these selenoproteins during *F. tularensis* infection.

Previous studies have shown Se through selenoproteins alter the phenotype of macrophages from proinflammatory M1 to anti-inflammatory M2 (32), *via* cyclooxygenase-dependent cyclopentenone prostaglandin J2 (90) or ‘eicosanoid class switching’ (74). Se supplementation promotes selenoprotein expression in macrophages that skews the arachidonic acid pathway from pro-inflammatory mediators prostaglandin E2 and thromboxane A2 to produce prostaglandin D2 and the downstream ant-inflammatory metabolites cyclopentenone prostaglandins (74). The beneficial effects of Se and selenoproteins of macrophages mediating an M2 immune response have been observed in mouse models of Se dietary deficiency and in Trsp^M^ mice during *N. brasiliensis* infection as this parasite requires a strong type 2 immune response for resolution of infection (27). Additional investigations utilizing Trsp^M^ mice to determine the contribution of macrophage specific selenoproteins to both bacterial and chemically-induced colitis revealed that the selenoproteins of macrophages are essential to clear *C. rodentium* and resolve inflammation. Trsp^M^ mice exhibited increased mortality as a result of *C. rodentium* infection (26), while Trsp^M^ were unable to resolve colitis-associated inflammation regardless if they were maintained on either Se-supplemented and deficient diets (25). While the role of M2 macrophages in tularemia pathogenesis is debated, anti-inflammatory M2 macrophages have not been previously shown to be protective against *F. tularensis*-induced pneumonic tularemia. In fact, classically activated M1 macrophages are thought to control *F. tularensis* (91, 92). Therefore, we believe the inability of Trsp^M^ and Se-deficient macrophages to control *F. tularensis* could be attributed to mechanisms other than their failure to differentiate into M2 macrophages.

Various selenoproteins have been demonstrated to have antioxidant functions and are critical in maintaining the redox status of the host cells. LPS stimulation of Se-deficient macrophages with correspondingly low selenoprotein expression elevated total cellular oxidative tone, nitric oxide synthase, and increased nitric oxide production, while Se-supplemented macrophages dampened reactive oxygen species (ROS) (71). Several studies have shown that IFN-γ activated macrophages restrict *F. tularensis* replication *via* a nitric oxide-dependent mechanism (93–95). *F. tularensis* also alters antioxidant defenses and proinflammatory cytokine production to promote intracellular survival (96). Therefore, it is tempting to speculate that selenoprotein-deficient macrophages have higher ROS levels leading to reduced bacterial growth. However, nitric oxide production was similar between WT or Trsp^M^ macrophages infected with *F. tularensis* (data not shown). In fact, it is known that *F. tularensis* neutralizes ROS/reactive nitrogen species by inhibition of NADPH oxidase within the resting phagocytes, thus promoting intracellular bacterial survival (97). In addition, the oxidative stress response system of *F. tularensis*, comprised of superoxide dismutases (98), and an H2O2-decomposing enzyme catalase (99), are necessary for intracellular growth and virulence. Therefore, we speculate that host selenoproteins must be restricting bacterial replication during pulmonary tularemia by a distinct mechanism.

Upon intracellular infection, *F. tularensis* becomes phenotypically auxotrophic and requires amino acids from the host for its survival and replication (100). In order to sequester nutrients from host cells, one of the strategies that the pathogen employs is the alteration of host autophagy (101). Dietary Se-deficiency induces autophagy as demonstrated through an upregulation of autophagy associated gene 5 and Beclin 1 mRNA and protein expression, and morphological changes in autophagy vacuoles, autolysosomes, and lysosomal degradation in the immune organs of chickens in comparison to Se diet controls (102). Since Se-adequate or supplemented macrophages are skewed toward an M2 phenotype (32) and have lower mTOR activation (103, 104), a kinase necessary for induction of canonical autophagy, selenoproteins in macrophages may be inhibiting autophagy induced by *F*. *tularensis*. However, autophagy is a complex process utilized by both the host and pathogen to facilitate survival, and the mechanism of macrophage selenoprotein regulation of autophagy during *F*. *tularensis* infection remains to be fully elucidated and is currently being investigated.

Herein, we identified a novel role for host macrophage selenoproteins to limit intracellular replication of a bacteria. Our studies could have direct implications for treating infectious diseases such as tuberculosis. In addition, these findings can have implications in managing diseases caused by infectious agents, such as sepsis. Sepsis is a pathophysiologic process involving activation and dysregulation of pro-inflammatory/anti-inflammatory responses intertwined with other physiological processes. Sepsis patients suffer organ damage from a dysregulated immune response to pathogen(s) that results in increased oxidative stress (105). Due to altered hepatic Se metabolism, sepsis patients exhibit decreased plasma Se concentrations as synthesis of the protein that transports Se, selenoprotein P (SelenoP), decreases in the liver and therefore less Se is transported throughout the body (106, 107). Accordingly, a beneficial role for Se supplementation was suggested for positive outcome by relieving oxidative stress and inflammation (108). In fact, several small-scale clinical studies have demonstrated a positive prognosis when Se was given as an adjunctive therapy (109, 110). Our findings provide additional evidence that Se supplementation may help during infectious disease particularly in infections by intracellular bacteria *via* inhibition of bacterial replication to control infection.

## Data Availability Statement

The datasets presented in this study can be found in online repositories. The names of the repository/repositories and accession number(s) can be found below: NCBI SRA; PRJNA732368.

## Ethics Statement

The animal study was reviewed and approved by The Pennsylvania State University Institutional Animal and Care Use Committee.

## Author Contributions

RM and KR planned and performed experiments, analyzed the data, supervised experiments performed by others, and wrote the manuscript. BK, SS, MN, SN, DW, DP, KD, AT, and AS performed some of the experiments and analyzed the data. SG and KP analyzed data and helped write the manuscript. BC provided critical reagents and helped write the manuscript. GK planned and supervised the study, procured funding, analyzed the data, and wrote the manuscript. All authors contributed to the article and approved the submitted version.

## Funding

RLM and DRW were supported by T32AI074551, SES by T32GM108563, MJN by TL1TR002016, and AT by T32LM012415 from the National Institutes of Health. KSP and GSK were supported by USDA-NIFA grant #4771 (Accession number 0000005). In addition, SG was supported by GM121907, KSP was supported by DK077152 and GSK was supported by NIH grants AI123521 and AI77917 from the National Institutes of Health.

## Conflict of Interest

The authors declare that the research was conducted in the absence of any commercial or financial relationships that could be construed as a potential conflict of interest.

## Publisher’s Note

All claims expressed in this article are solely those of the authors and do not necessarily represent those of their affiliated organizations, or those of the publisher, the editors and the reviewers. Any product that may be evaluated in this article, or claim that may be made by its manufacturer, is not guaranteed or endorsed by the publisher.
