# Supplementary material for: Macrophage Selenoproteins Restrict Intracellular Replication of Francisella tularensis and Are Essential for Host Immunity
Source: Front Immunol. 2021 Oct 29;12:701341. doi: 10.3389/fimmu.2021.701341 (PMC8586653; doi:10.3389/fimmu.2021.701341)
Supplement: Supplementary file 1 [file DataSheet_1.docx]

***Supplementary Material***

**Supplementary Methods**

**Trsp^M^ genotyping**

Genotyping was performed on transgenic animals to verify successful deletion of the *Trsp* gene as previously described (1). Briefly, ear tissue samples from all mice were placed in 200µl tail lysis buffer (50mM Tris-HCL, 25mM EDTA, 50mM KCL and 0.4% (v/v) NP40 Tween-20) with a 1:50 (v/v) OB Protease Solution (Omega Bio-Tek, GA, USA) and incubated at 55ºC overnight. Lysates were vortexed, incubated at 95ºC for 5 min and centrifuged at 10,000 × *g* for 2 min at RT. Supernatants were used for PCR, which was prepared with 5X GoTaq® Buffer (Promega, WI, USA), 1 µM MgCL_2_ (New England BioLabs, MA, USA), 200µM dNTPs (New England BioLabs) 0.20 µM of each primer 1U of GoTaq® DNA polymerase, and 1 µl of DNA lysate. Samples were amplified with the program of 95ºC/5min (1 cycle), 95ºC/0.5min, 59 ºC for Trsp or 54 ºC for LysM/1min, 72 ºC/1min (40 cycles) followed by a final 72 ºC/3min extension. Products were run on an 1.8% agarose gel, imaged with a Gel Doc™ (Bio-Rad, Universal hood II, USA) and Image Lab software (Bio-Rad, version 5 build18). The primers used to detect regions of interest and product sizes are provided in Table S3.

**Se status confirmation by GPX1 Western blot**

BMDMs from Se deficient, adequate or supplemented diet mice were lysed with RIPA buffer plus 1X HALT™ Protease Inhibitor cocktail- 1X EDTA solution (ThermoFisher Scientific, USA) for 10 min followed by dilution with 2X laemmli buffer (Biorad, USA). MiniProtean® TGX™ precast gels (Biorad, USA) were used to separate proteins that were then transferred to PVDF membranes using the Biorad Turbo Blot system and Trans-Blot® Turbo™ transfer packs (Biorad, USA).  Blots were blocked in 5% fetal bovine albumin (Sigma-Aldrich, USA) and incubated in 1:1000 rabbit anti-mouse GPX-1 (Abcam, USA) or 1:500 GPX-4 (ABclonal Science, Inc., Cn) and 1:100,000 rabbit anti-mouse β-actin. Blots were washed incubated with a goat anti-rabbit H&L chain secondary antibody conjugated to horseradish peroxidase enzyme at a dilution of 1:7500. The blots were developed with horseradish peroxidase substrate (EMD Millipore, Germany) and imaged and analyzed with a Gel doc (Bio-Rad, Universal hood II) and Image Lab software (Bio-Rad, version 5 build18).

**qPCR selenoprotein analysis**

Gentamicin protection assays were performed with WT BMDMs at an MOI=0.1 using *F. tularensis* LVS (n=3). Cells were collected at 18hr p.i. and processed for RNA isolation using a combination method incorporating both TRIzol reagent and a Purelink™ RNA Mini Kit (Invitrogen, Carlsbad, CA). An iScript™ Reverse Transcription Supermix (Bio-Rad, Hercules, CA) was used for RT-PCR to generate cDNA, and SsoAdvanced Universal SYBR Green Supermix (Bio-Rad, Hercules, CA) was used to perform qPCR with a CFX96 Touch Real-Time PCR Detection System. Analysis was done using CFX Manager™ Software. mRNA expression was determined using the 2**^-ΔΔCT^**, where the change in the cycle threshold (C_T_) values are compared to the expression of uninfected BMDM controls and then normalized to the expression of reference gene *18s rRNA.*

**Statistical Analysis**

Statistical significance was assessed for gentamycin protection assay with *F. tularensis* LVS grown in the absence or presence of Se using an unpaired t-Test with Welch’s correction (p<0.05).

**Supplementary Table 1**. Bacterial virulence gene primers for qPCR

| **Gene** | **Forward** | **Reverse** |
| --- | --- | --- |
| FTL _1666,  *Pol*A | 5’-TACACGACCATTTGCGTCCA-3’ | 5’-CAGCAAAGGGTCAAGTGTCG-3’ |
| FTL _0421,  *Tul*4 | 5’-GTGCCATGATACAAGCTTCC-3’ | 5’-GCTGTCCACTTACCGCTTCA-3’  5’-GCTGTCCACTTACCGCTACA-3’ |
| FTL _1159,  *Igl*C | 5’CCAGGCTCTATAAATCCAACAATA-3’ | 5’-TTTCATATCTGTAGCACTTGCTTG-3’ |
| FTL _1328,  *Fop*A | 5’-AGGATCTGTTCAAGGTGCTT-3’ | 5’-GCATTAGGCTGGTTCATTCC-3’ |

Abbreviations: *PolA*: DNA polymerase (reference number: FTL _1666), *Tul4*: 17kDa major membrane protein (FTL _0421), *IglC*: Intracellular growth locus, subunit C (FTL _1159) and *FopA*: OmpA family protein (FTL_1328)

**Supplementary Table 2.** *F. tularensis* LVS virulence gene expression is not altered by the presence of Se in liquid culture.

| **Virulence Gene** | **2^-ΔΔCT^** | **SD** | **Significance** |
| --- | --- | --- | --- |
| *FopA* | 1.11 | 0.11 | ns |
| *IglC* | 0.91 | 0.51 | ns |
| *Tul4* | 0.62 | 0.36 | ns |
| **Control Gene** |  | | |
| *PolA* | 1 |  | |

mRNA values are expressed as 2**^-ΔΔCT^**, where the change in the cycle threshold (C_T_) values are compared to the expression of Se-deficient controls and then normalized to the expression of reference gene *PolA*. Abbreviations: *FopA*: OmpA family protein (reference number: FTL_1328), IglC: Intracellular growth locus, subunit C (FTL _1159) and *Tul4*: 17kDa major membrane protein (FTL _0421) *PolA*: DNA polymerase (FTL _1666).

**Supplementary Table 3.** Log fold change (logFC) and false discovery rate (FDR) values for 20 differentially expressed selenoproteins between BMDMs uninfected or infected with *F. tularensis*.

| **Gene** | **logFC** | **FDR** |
| --- | --- | --- |
| Selenow | 0.89317 | 0.011281 |
| Gpx1 | 0.727792 | 0.013537 |
| Selenom | 0.630829 | 0.016977 |
| Gpx4 | 0.635538 | 0.02305 |
| Msrb1 | 0.555449 | 0.034423 |
| Selenoh | 0.587377 | 0.039061 |
| Selenop | -0.388692 | 0.052651 |
| Selenok | 0.22034 | 0.216985 |
| Selenot | -0.198137 | 0.277833 |
| Dio2 | -0.882099 | 0.286429 |
| Txnrd2 | 0.227441 | 0.301086 |
| Selenoi | -0.195903 | 0.309784 |
| Txnrd3 | 0.285105 | 0.402086 |
| Sps2 | 0.161195 | 0.408298 |
| Gpx3 | 0.175634 | 0.583768 |
| Txnrd1 | -0.10199 | 0.611495 |
| Selenoo | 0.061603 | 0.816241 |
| Selenos | -0.043135 | 0.8642 |
| Selenof | -0.028899 | 0.902551 |
| Selenon | -0.004251 | 0.98546 |

Abbreviations: Dio2: type II iodothyronine deiodinase; GPX: glutathione peroxidase; Msrb1: methionine sulfoxide reductase B1; Selenof: selenoprotein F; Selenoh: selenoprotein H; Selenoi: selenoprotein I; Selenok: selenoprotein K; Selenom: selenoprotein M; Selenon: selenoprotein N; Selenoo: selenoprotein O; Selenop: selenoprotein P; Selenos: selenoprotein S; Selenot: selenoprotein T; Selenow: selenoprotein W; Sps2: selenophosphate synthetase-2; Txnrd: thioredoxin reductase.

| Gene | Sequence | Product | |  | |
| --- | --- | --- | --- | --- | --- |
| Trsp CKNO2 F’ | 5’-GCAACGGCAGGTGTCGCTCTGCG-3’ | | **Trsp^wt/wt^ = 900 bp**  **Trsp^fl/f l^= 1100 bp**  **Trsp^wt/fl^ = 900 bp &1100 bp** | **** |  |
| Trsp CKNO8RPR’ | 5’-CGTGCTCTCTCCAGTGGCTA-3’ | |  |  |  |
| MLys1 F’ | 5’-CTTGGGCTGCCAGATTTCT-3’ | | **LysM^wt/wt^ = 350 bp LysM^Cre/Cre^ = 700 bp**  **LysM^wt/cre^=700 bp &350 bp** | **** |  |
| MLys2 R’ | 5’-TTACAGTCGGCCAGGCTGAC-3’ | |  |  |  |
| LysM Cre8 R’ | 5’-CCCAGAAATGCCAGATTACG-3’ | |  |  |  |

**Supplementary Figure 1. Trsp^M^ genotyping primers and products.**

A

B

**Supplementary Figure 2. Se supplementation does not alter the growth kinetics of *F. tularensis* LVS in liquid culture.** Growth of *F. tularensis* LVS was monitored for 24 hr in CDM under various Se conditions (red=Se deficient (0 nM), black=Se adequate (50 nM), blue= Se supplemented (200 nM)) by measuring (A) OD_600nM_ and (B) CFU recovery at indicated times. Data are representative of two *in vitro* experiments, error bars in (A) represent +/- SD, n=3/ group.

**Supplementary Figure 3. The presence of Se in *ex vivo* culture does not affect the entry and replication of *F.*** ***tularensis* LVS.** BMDMs from 6-8 week old C57BL/6 mice were generated in Se deficient conditions. BMDMs were infected with *F. tularensis* LVS at an MOI of 1:100 under Se deficient (0 nM Na_2_SeO_3_, red circle) or Se supplemented (200 nM Na_2_SeO_3_, blue triangle) culture conditions. (A) Entry at 2 hr and (B) intracellular growth of bacteria 24 hr post-infection were enumerated. The number of bacteria recovered at 2 and 24 hr represented as the mean of replicates. Data are representative of three *in vitro* experiments. Statistical significance was assessed using an unpaired t-Test with Welch’s correction (p<0.05).

**Supplementary Figure 4. Se supplementation of *F. tularensis* LVS does not affect lung colonization during acute time points of infection.** *F. tularensis* LVS was grown in deficient (red circle) or supplemented (200 nM Na_2_SeO_3_) (blue triangle) culture conditions and 6–8 week-old C57BL/6 mice were inoculated with 10,000 CFU by intranasal route. Pulmonary bacterial burden was measured at 6, 12 and 24 hr post-inoculation. Bar represents the mean, *n*=3 mice/ group. A Two-way ANOVA was performed to assess statistical significance (p>0.05). Data are representative of two *in vivo* experiments.

**Supplementary Figure 5. Confirmation of Se status of diet mice by measuring BMDM GPX1 protein expression.** Six-to-eight week old C57BL/6 mice were maintained on Se deficient, adequate or supplemented diets for a minimum of 12 weeks. BMDMs were prepared and maintained *ex vivo* under Se deficient, adequate or supplemented conditions. Lysates were prepared and relative expression of GPX1 by western blot analysis is depicted with four technical replicates per diet group (A). Data are representative of three experiments.

**Supplementary Figure 6.** **Methylseleninic acid (MSA) and Na_2_SeO_3_ restricts *F. tularensis* LVS replication in BMDMs that may be due to an increase in selenoproteins as measured by glutathione peroxidase-1 expression.** Four week-old C57BL/6 mice were placed on Se deficient or supplemented diets and maintained for a minimum of 12 weeks. BMDMs were prepared and maintained *ex vivo* under deficient (0 nM) or supplemented (200 nM) conditions with MSA, selenomethionine (SeMet) or Na_2_SeO_3._ (A) BMDMs were infected with *F. tularensis* LVS at an MOI of 1:100 and intracellular growth of bacteria 24 hr post-infection were enumerated. Bacteria recovered at 24 hr are represented as the mean + SD of triplicates. (B) Lysates were prepared and expression of GPX-1 was measured by western blot analysis. GPX-1 expression is denoted as a ratio of GPX-1 to the internal control β-actin.

**Supplementary Figure 7. GPX1 expression is absent in Trsp^M^ macrophages.** BMDMs were isolated from WT or Trsp^M^ mice and maintained *ex vivo* under Se deficient conditions. Lysates were prepared and expression of GPX-1 was measured by western blot analysis in three technical replicates. GPX-1 expression is denoted as a ratio of GPX-1 to the internal control β-actin and data are represented as the mean + SD.

**Supplementary Figure 8. Trsp^M^ and WT mice succumb to *F.*** ***tularensis* LVS challenge with a greater inoculating dose**. Six-to-eight week-old WT and Trsp^M^ mice (n= 8-16/group) were intranasally inoculated with (A, B) 1500 CFU or (C, D) 1750 CFU of *F. tularensis* LVS and survival (A, C) body weight (B, D) were monitored daily for 14 days. Mice that lost greater than 20% body weight were euthanized. Significance of survival rate was assessed with the Log-rank (Mantel-Cox) test (*p <0.05 Trsp^M^ vs. WT, inoculating dose= 1500 CFU). Weights are represented as the mean % body weight of the surviving animals and error bars denote +/-SD.


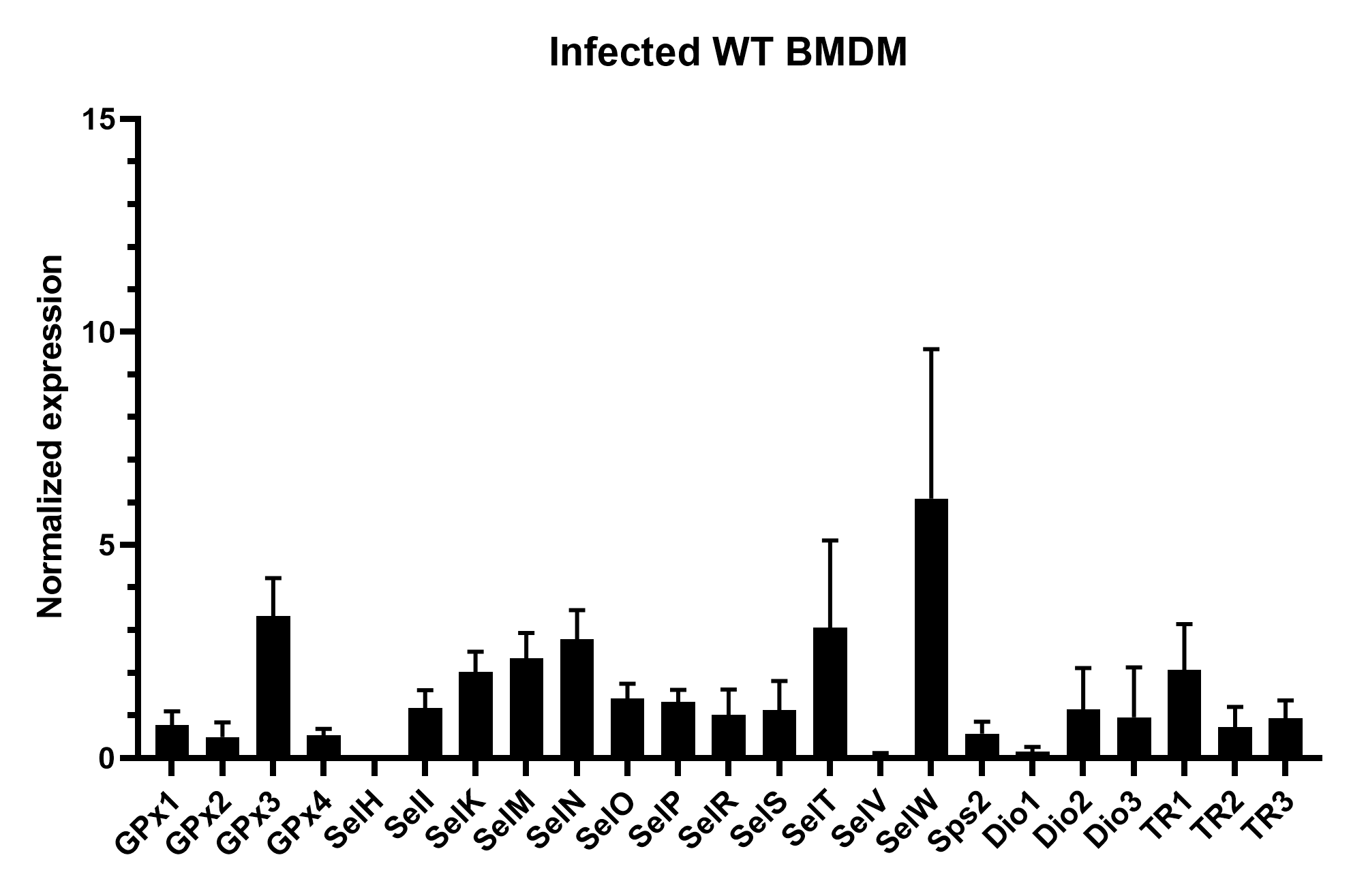


**Supplementary Figure 9. mRNA expression of selenoproteins in BMDMs infected with *F. tularensis* LVS.** Gentamicin protection assays were performed with WT BMDMs at an MOI=0.1 using *F. tularensis* LVS (n=3). Cells were collected at 18hr p.i., RNA isolation was isolated and subjected to qPCR. mRNA expression is depicted as 2**^-ΔΔCT^**, where the change in the cycle threshold (C_T_) values are compared to the expression of uninfected BMDM controls and then normalized to the expression of reference gene *18s rRNA.* Error bars denote +SD.

**Supplementary Figure 10. Selenoprotein protein expression** **in BMDMs infected with *F. tularensis* LVS.** Gentamicin protection assays were performed with WT BMDMs at an MOI=0.1 using *F. tularensis* LVS (n=3). Cells were collected at 10 hr p.i., lysates were prepared, and expression of (A) GPX-1 or (B) GPX-4 was measured by western blot analysis in three technical replicates. Expression is denoted as a ratio of GPX-1 or GPX-4 to the internal control β-actin and data are represented as the mean + SD.

Reference:

1. Nelson SM, Shay AE, James JL, Carlson BA, Urban JF, Jr., Prabhu KS. 2016. Selenoprotein Expression in Macrophages Is Critical for Optimal Clearance of Parasitic Helminth Nippostrongylus brasiliensis. *J Biol Chem* 291: 2787-98
